# Reducing worry and rumination in young adults via a mobile phone app: study protocol of the ECoWeB (Emotional Competence for Well-Being in Young Adults) randomised controlled trial focused on repetitive negative thinking

**DOI:** 10.1186/s12888-021-03536-0

**Published:** 2021-10-21

**Authors:** Daniel Edge, Alexandra Newbold, Thomas Ehring, Tabea Rosenkranz, Mads Frost, Edward R. Watkins

**Affiliations:** 1 Mood Disorders Centre, School of Psychology, University of Exeter, Exeter, EX4 4LN UK; 2 Department of Psychology, LMU, Munich, Germany; 3 Monsenso ApS, Copenhagen, Denmark

**Keywords:** Depression, Well-being, Young people, Mobile-health, Prevention, Randomised controlled trial, Emotional competence, Rumination, Worry, Cognitive behavioral therapy

## Abstract

**Background:**

Promoting well-being and preventing poor mental health in young people is a major global priority. Building emotional competence skills via a mobile app may be an effective, scalable and acceptable way to do this. A particular risk factor for anxiety and depression is elevated worry and rumination (repetitive negative thinking, RNT). An app designed to reduce RNT may prevent future incidence of depression and anxiety.

**Method/design:**

The Emotional Competence for Well-Being in Young Adults study developed an emotional competence app to be tested via randomised controlled trials in a longitudinal prospective cohort. This off-shoot study adapts the app to focus on targeting RNT (worry, rumination), known risk factors for poor mental health. In this study, 16–24 year olds in the UK, who report elevated worry and rumination on standardised questionnaires are randomised to (i) receive the RNT-targeting app immediately for 6 weeks (ii) a waiting list control who receive the app after 6 weeks. In total, the study will aim to recruit 204 participants, with no current diagnosis of major depression, bipolar disorder or psychosis, across the UK. Assessments take place at baseline (pre-randomisation), 6 and 12 weeks post-randomisation. Primary endpoint and outcome for the study is level of rumination assessed on the Rumination Response Styles Questionnaire at 6 weeks. Worry, depressive symptoms, anxiety symptoms and well-being are secondary outcomes. Compliance, adverse events and potentially mediating variables will be carefully monitored.

**Discussion:**

This trial aims to better understand the benefits of tackling RNT via an mobile phone app intervention in young people. This prevention mechanism trial will establish whether targeting worry and rumination directly via an app provides a feasible approach to prevent depression and anxiety, with scope to become a widescale public health strategy for preventing poor mental health and promoting well-being in young people.

**Trial registration:**

ClinicalTrials.gov, NCT04950257. Registered 6 July 2021 – Retrospectively registered.

## Background

There is a growing concern about the high, and steadily increasing, rates of poor mental health in young people and the early onset of mental disorders such as depression and anxiety [1]. Poor mental health during this formative period in a young person’s life can severely affect their future life chances, with a significant long-term impact on health, education, employment and social outcomes [1–4]. The incidence of depression and anxiety both markedly increase through mid-adolescence and peak during young adulthood [2]. For this reason, there has been a call for urgent improvement in primary prevention of poor mental health as well as promoting mental well-being in young people [4, 5].

While evidence-based primary prevention interventions for common mental health disorders already exist, systematic reviews suggest that effect sizes are relatively small [6–9]. Most evidence-based interventions also require considerable person-hours from professionals, such as the involvement of teachers and therapists. Ideally, we require mental health promotion and prevention approaches that are both more effective and that can be delivered simultaneously to many users (i.e., non-consumable). Such approaches would not need input from practitioners, and thus will be nearly unlimited in scale, making them suitable to be used as a public health approach at a population level.

The Assessing and Enhancing Emotional Competence for Well-being in the Young (ECoWeB) project tries to tackle these challenges by integrating the use of a smartphone application with an intervention based on a model of normal emotional functioning [10]. The use of mobile apps (sometimes called Behavioural Intervention Technologies, BITs) [11] has a number of potential advantages: (i) scalability - Mobile-health (m-health) technologies are highly scalable, allowing very good coverage and reach; they are widely accessible; (ii) non-consumable – they enable repeated use by nearly unlimited people simultaneously; (iii) convenience – they can be used anytime, anywhere; (iv) acceptability – mobile apps are highly used by young people, with the majority of young people using smart phones [12]. In addition, mobile apps can help integrate behavioural changes into daily life: the app is always on hand via the smartphone, making it well-suited for changing habits. Despite a huge increase in the number of m-health apps (> 10 k) [13], only a very small minority have been based on robust science, utilised established treatment principles and been rigorously tested with respect to safety and efficacy in robust well-powered randomised controlled trials (RCTs) [14–17]. There is emerging evidence that m-health apps can deliver efficacious treatment interventions for anxiety and depression [18, 19], although few trials have examined well-being promotion and prevention of poor mental health in young people specifically.

Rather than traditional clinical disease models of psychopathology, the ECoWeB project adopts an approach to promoting mental health based on an established theoretical model of normal emotional functioning – the Component Process Model of Emotion (CPM) [20–22]. This model proposes that individuals vary in their abilities across different areas of Emotional Competence (EC), including: (i) accurate and functional appraisals of emotional situations and of the individual’s ability to cope with these situations, which determines whether an individual experiences the emotion appropriate to a situation (Emotion Production); (ii) abilities to perceive and understand emotions in themselves and others (Emotion Knowledge and Perception); (iii) and the use of more adaptive versus less adaptive strategies to manage and regulate emotions (Emotion Regulation), for example, reducing worry and rumination. The model hypothesizes that good EC functioning contributes to reduced anxiety and depression, and improved mental well-being. Considerable correlational and prospective data is consistent with this hypothesis [23–28]. As such, the treatment app for the main study has been developed with psychoeducation, learning exercises and self-help tools designed to tackle each of these distinct elements, personalised to each individual [10]. The current study tests the usefulness of one of those components alone as a single-intervention app: targeting worry and rumination in individuals with elevated worry and rumination.

Worry and rumination have a strong correlation with each other and share many characteristics, which means they are often referred to under the unitary construct of Repetetive Negative Thinking (RNT) [29–31]. RNT processes, such as worry and rumination, are known proximal risk factors implicated in the onset, maintenance and relapse of depression and anxiety, which have been identified as malleable targets for intervention [32, 33]. Prospective longitudinal research has demonstrated that degree of rumination predicts (a) onset and duration of major depressive episodes [34, 35]; (b) depressive symptoms, after controlling for baseline depression and anxiety across a range of follow-up periods [36, 37]; and (c) slower response to Cognitive-Behavioural Therapy (CBT) and antidepressant treatment, as well as reduced likelihood of recovery following treatment [38, 39]. Degree of rumination also mediates the effects of other identified risk factors such as neuroticism, past history of depression, family history of poor mental health and stressful life events on depressive onset [40]. Importantly, rumination is a sensitive, relevant risk factor for depression in young people and prospectively predicts fluctuations in depressive symptoms over time in this age group [31, 33, 34, 41]. Similarly, research has shown that increased levels of worry are predictive of both anxiety and depressive symptoms [42] and that worrying on a daily basis predicts increases in daily anxiety [43]. Worry has also been found to be strongly associated with symptoms of anxiety, depression and prolonged grief, both longtitudinally and concurrently [44]. There is also experimental evidence for the induction of rumination and worry exacerbating symptomatology related to anxiety and depression such as negative thinking, poor problem solving, negative affect and delayed decision-making speed [45–48].

The ECoWeB app under investigation in this study adapts a proven intervention that targets a shift away from maladaptive RNT (worry and rumination), to more adaptive problem-solving. This RNT focused self-help intervention builds on proven cognitive-behavioural therapy principles and includes identifying warning signs for worry and rumination, repeated practice to train out of unhelpful habits and build helpful habits, and the training of useful alternative strategies such as being more specific, relaxation, problem-solving and self-compassion [49]. This intervention has been proven to be effective in reducing and preventing depression and anxiety in face-to-face therapy [50–52] and in web-based interventions for young adults [53] including an entirely self-help variant [54].

Prevention mechanism trials [55] seek to establish whether interventions can reduce specific risk factors for psychopathology or increase established resilience factors. Since worry and rumination are established specific risk factors, this trial is a prevention mechanism trial to test whether use of an app targeting RNT can reduce worry and rumination in young people, and thus, has potential as a preventative intervention, i.e., a proof-of-concept study of the potential of the RNT-targeting app.

## Objective

The primary objective of this phase III prevention mechanism randomised controlled trial is to evaluate whether digital EC self-help targeting RNT is effective at reducing self-reported rumination and worry at 6-week follow-up in young people, relative to usual practice waiting list control. Self-reported worry and rumination are potential predictors of risk for future depression and anxiety, that is, this operates as a targeted trial of a prevention mechanism [55, 56].

Secondary objectives are to examine the efficacy of the app secondary outcomes including symptoms of anxiety and depression and well-being at 6 weeks and 12 weeks.

## Methods

The study will be conducted and reported according to Consolidated Standards of Reporting Trials (CONSORT) [57, 58] and extensions for non-pharmacologic treatment interventions and multi-arm parallel-group randomised trials and CONSORT-EHEALTH for improving and standardising evaluation reports of Web-based and mobile health interventions [59].

### Study design

The trial design is a superiority two-arm parallel-group single-blind randomised controlled trial comparing usual practice plus up to 6 weeks of using the RNT-targeting digital self-help app versus usual practice and waiting list control. The analysis team members will be blind to the treatment arm. Our primary hypothesis is that the RNT-targeting digital self-help app will reduce rumination and worry significantly more than waiting list control at 6 weeks follow-up.

Potential participants provide initial consent to complete screening measures to determine if they are eligible to participate. Any potential participants who are found not to be eligible are automatically signposted to other sources of support. Once trial eligibility has been determined and consent to participate in the trial has been obtained, participants are individually selected at random (in a 1:1 ratio) to be immediately offered self-help components within a mobile phone app to target worry and rumination or to wait 6 weeks before being offered self-help components within a mobile phone app to target worry and rumination. Thus the two trial arms are: [1] waiting list control; [2] digital CBT self-help including specific intervention elements to target worry and rumination.

### Recruitment and study settings

We seek to recruit 204 young adult participants within the United Kingdom. The recruitment strategy includes online and website advertising; email to mailing lists; newsletters and other circulars and noticeboards within willing schools, colleges and universities. A social media campaign will also be designed and prepared to be carried out on different social networks (e.g., Facebook, YouTube, Instagram, Twitter).

### Outcomes

Outcomes will be assessed at baseline (pre-randomisation) and 6 weeks and 12 weeks post-randomisation.

### Primary outcome

The primary outcome measure will be the 22- item Ruminative Response Scale (RRS) [36, 60], a well-established measure of pathological rumination, which predicts subsequent depression, at 6 week follow-up (the primary endpoint).

### Secondary outcomes

Secondary outcomes include change between baseline, 6 weeks and 12 weeks post randomisation on the following: the Penn State Worry Questionnaire (PSWQ) [61], a well-validated 16-item measure of trait tendency towards worry; symptoms of depression as assessed on the Patient Health Questionnaire-9 (PHQ-9) [62], a well-validated measure of depression; 14-item Warwick-Edinburgh Mental Well Being Scale (WEMWBS) [63, 64], a leading validated self-reported index of well-being with excellent psychometric properties; The Generalized Anxiety Disorder-7 (GAD-7) questionnaire will be used to assess anxiety symptoms [65]. A further secondary outcome will include changes between 6 weeks and 12 weeks post randomisation measured on the RRS.

The following descriptive variables will be assessed only at baseline: age, gender, ethnicity, employment status and historical diagnosis of a mental health condition.

### Eligibility criteria

Eligible participants will be: [1] young people aged 16 to 24 years old, [2] based in the UK, [3] having basic literacy in English, [4] able to provide informed consent, [5] having self-identified concerns about worry and rumination and elevated levels of RNT, defined here as scoring above the 50th percentile (i.e., top-half of scale) on either the RSS (> 34) or the PSWQ (> 41); and [5] having regular access to a smart phone (android or iOS) (see Table 1).

**Table 1:** Inclusion and exclusion criteria

**Inclusion criteria**	
16 to 24 years old	
Living in the UK	
Regular access to Android or iOS smartphone	
Reports a high level of rumination and/or worry	
**Exclusion criteria**	
Current diagnosis of Clinical depression, bipolar disorder or psychosis	
Current use of medication or psychological interventions to treat a mental health problem	
Current suicidality^1^	

Participants will be excluded from the trial at baseline if presenting with highly elevated symptoms of depression indicating more specialist treatment is required (PHQ-9 > 20). Other exclusion criteria include: active suicidality, currently receiving psychological therapy, counselling or psychiatric medication including antidepressants.

### Screening and consent procedure

Potential participants who are interested in the study are directed to our study website, (www.mymoodcoachworry.co.uk), which provides further information, including eligibility criteria, and a pre-screener to check their age. If appropriate, the website visitor is provided with the study information sheet and an initial consent screen to provide contact details (email; mobile phone number), and to provide informed consent to complete the baseline questionnaires. Individuals who are not suitable at pre-screen (e.g., outside of age range) will automatically be directed to a webpage explaining why they are not suitable for the trial. Those reporting mental health difficulties will be automatically guided to webpages providing information, guidance including to consult with their general practitioner (or equivalent), and weblinks and telephone numbers for help and support, including contact details for the trial team.

After pre-screening, potential participants are provided with a copy of the information sheet, privacy policy and consent form so they can be reviewed prior to giving any consent. Once this initial consent is provided, the participant proceeds to the baseline assessment.

Those meeting eligibility criteria following the baseline assessment are then asked to consent to take part in the trial, using an electronic information sheet, consent form and electronic signature. Once eligible participants consent to participate in the trial, they will be randomised into one of the two conditions.

### Participant timeline (see Figures 1 and 2)

<Insert SPIRIT diagrammatic schedule and CONSORT diagram here>.

### Baseline and follow-up assessments

The baseline assessment takes place after initial electronic informed consent is provided and consists of web-based self-report measures to assess worry and rumination, current well-being, symptoms of anxiety and depression (see outcome measures and Table 2).

**Table 2:** Measurements and Endpoints

			Follow-up (weeks)	
Web Assessment		Baseline	6	12
**Pre-screening**	date of birth, self-reported mental health	✓		
**Informed Consent**		✓		
**Socio-demographics**	Age, sex, employment status, ethnicity, historic mental health problems	✓		
**Rumination**	RRS questionnaire; primary outcome	✓	✓	✓
**Worry**	PSWQ questionnaire; secondary outcome	✓	✓	✓
**Well-being**	WEMWBS questionnaire; secondary outcome	✓	✓	✓
**Depression**	PHQ-9 questionnaire; secondary outcome	✓	✓	✓
**Anxiety**	GAD-7 questionnaire; secondary outcome	✓	✓	✓
**Feedback about use of app and experience**				✓

**Fig. 1:**
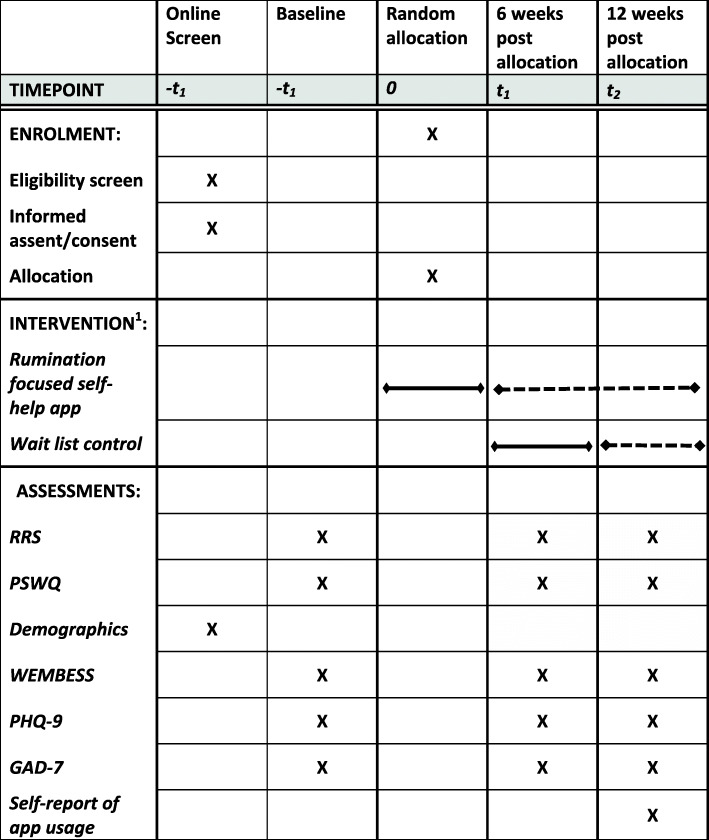
Schedule of enrolment, interventions, and assessments

All participants are entered into the trial and followed up electronically at 6 and 12 weeks post-randomisation. All assessments will be routinely collected online using the assessment website following automated reminders, without the involvement of researchers: At each follow-up point, participants will be automatically sent emails with links to enter their data into the assessment website. All participants will be prompted to complete follow ups via email and text message if they do not respond to the automated reminders. Site researchers involved in collecting follow-up data will be blind to treatment allocation and separate from other team members that are available to participants to follow-up on risk and answer technical queries. Any unblinding in contact with a site researcher would be logged as protocol violations and only a researcher that has remained blind will be able to prompt future follow-up from that participant. Figure 1 and Table 2 give an overview of all measurements.

**Fig. 2:**
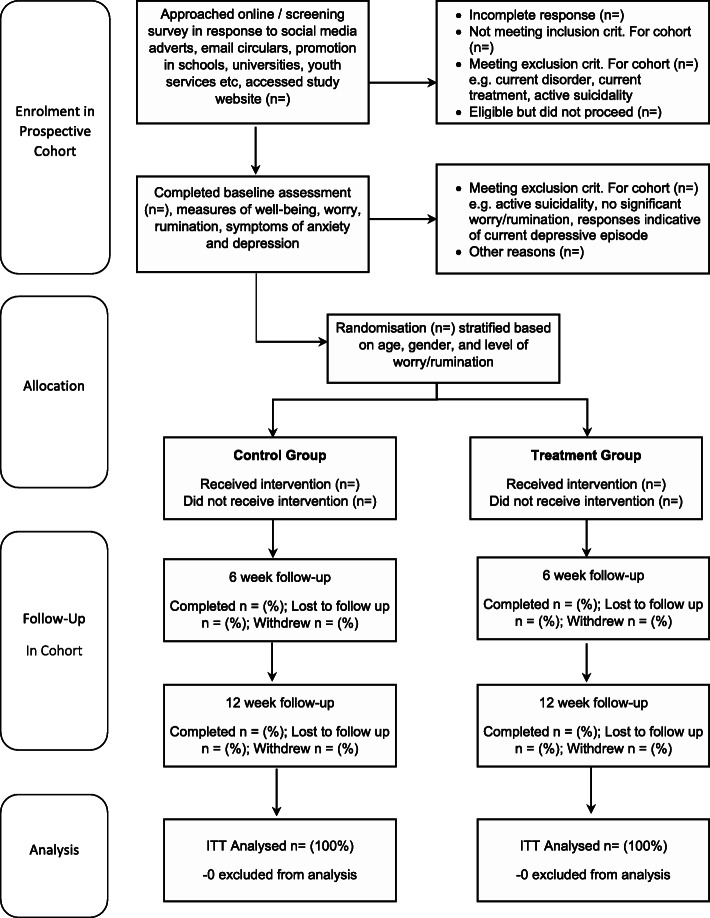
CONSORT flow diagram

### Randomisation, intervention delivery and masking

Participants will be randomised (in a 1:1 ratio) to the two intervention arms. Randomisation will be conducted independently using pre-generated computerised allocations. To promote balance across key participant characteristics across intervention arms, randomisation will be stratified according to gender (male, female, non-binary) and level of worry and rumination (higher, moderate). Prior studies found that individuals scoring in the worst quartile on measures of EC (e.g., rumination, appraisals, interpersonal vulnerabilities) have elevated risk for subsequent anxiety and depression [66]. Elevated levels of RNT have previously been identified for individuals scoring in the worst quartile (at or above 75th percentile) on at least one measure of worry (PSWQ) and rumination (RRS) and in the top tercile on the other measure (at or above the 66th percentile) [53]. We therefore wanted to stratify participants self-identifying as worriers into those who met this threshold criteria (higher RNT group), and those who did not meet this criteria (moderate RNT group, i.e., at least one measure scoring between 50th and 75th percentile). Consenting participants in the treatment group will then be signed up to use the RNT focused variant of the app by members of the research team not involved in assessment. The participant’s email address is used to provide them access to the app.

### Interventions

#### Digital RNT- targeting self-help app (experimental intervention group)

The self-help app includes self-monitoring, psychoeducation and active self-help exercises. The self-monitoring includes daily mood ratings and an ecological momentary assessment option (MoodTracker) for more detailed analysis of mood, worry, activity and situational context. This self-monitoring is intended to help young people learn more about their emotional experiences and what influences them, and to spot the relationship between where they are, who they are with, what they are doing and their worry and rumination. The digital self-help provides psychoeducation, tips, advice, exercises and training for each individual focused on reducing RNT, using strategies from the proven rumination-focused CBT intervention [50–54]. The app includes text, pictures, audio-recordings, animations, audio-exercises to practice (e.g., self-compassion, relaxation, concreteness exercises), and questionnaires with tailored feedback. It features a menu structure including a dashboard to monitor notifications and progress, an explore function to graph the self-monitoring responses made by the participant, Challenges that provide learning exercises, and Tools that are brief strategies that young people can use in the moment when they need them. For example, the Tools for tackling rumination and worry include self-compassion, learning to think in a more concrete way to promote problem-solving, relaxation, and breathing exercises. The app is designed for iOS and Android use.

#### Waiting list control

The waiting list control group will receive the RNT-targeting digital self-help app after a six week wait.

#### Intervention adherence

The use of the app will be assessed and recorded including number of times the app is used. A minimum intervention dose for the treatment group (treatment compliance) will be defined a priori, based on the principle that users will benefit from self-monitoring, learning new ideas (completing Challenges) and practising new skills (completing Tools).

#### Sample size

The sample size was calculated based on an estimated minimum clinically important difference (MCID) for the primary outcome of RRS. Prior studies using the RRS indicated a standard deviation (SD) of 8 as the most conservative estimate for a normative population in the target age group (SD = 7.58–8.51) [53]. Combining consensus from experts in rumination, with one recommended approach to identifying a MCID as being half of the SD for the respective index [67], a proposed MCID for the RRS is a reduction of 4 points. Using these values for 90% power, with an alpha of 0.05, an online power calculator for continuous outcome superiority trials [68]^1^ indicated that 85 participants per group (170 in total) are required. Allowing for a 20% follow-up attrition rate, the estimated total sample size required would be 204 participants (102 per group).

### Statistical analysis plan

Statistical reporting will follow CONSORT standards [57]. Missing data will be inspected and handled via full information maximum likelihood (FIML) or multiple imputations (MI) as appropriate. The primary analyses will be intention-to-treat (ITT) analyses [69] (i.e. all participants will be included in the analyses according to their randomised allocation) and based on complete case outcome data. A full, detailed analysis plan, including plans for any interim analysis, subgroup analysis, and sensitivity analysis of the primary outcomes, will be prepared and finalised before the analysis. The primary outcome is change in rumination (RRS) from baseline to 6 weeks follow-up. Our analysis will be an Analysis of Covariance (or equivalent regression or linear mixed model) for the primary and secondary endpoints, with baseline scores as the covariate. Models will be fitted using generalized linear mixed models equivalent to ANCOVA regression models.

As a secondary analysis, sensitivity analyses will compare results of imputed models to primary analysis of complete case ITT models.

### Organization, quality assurance and data management

Research data will be automatically collected in a pseudonymised manner through an electronic data capture system delivered from the website through to the central study database. In the first instance all participants will be directed to the website to provide their data. All data will be kept securely and confidentially and only accessed by members of the research team.

### Trial status

The Trial was registered in ClinicalTrials.gov. Number of identification: NCT04950257 (www.clinicaltrials.gov).. Recruitment commenced in May 2021.

## Discussion

Improving the mental health of young people has recently been identified as a global health priority [4, 70]. This includes both the prevention of poor mental health, such as the onset of anxiety and depression, and the promotion of increased well-being. In order to reach large numbers of young people, effective approaches to improve prevention and well-being promotion need to be widely accessible and highly scalable. As the majority of young people use mobile devices, one potential approach to delivering such an intervention is through the use of mobile apps (m-health).

The identification of effective prevention interventions can be achieved by developing interventions that target known mechanisms for vulnerability to mental health disorders and evaluating whether they effectively reduce this vulnerability factor using prevention mechanism trials [55]. This trial is one such prevention mechanism trial and will thus test as proof-of-concept whether the self-help app is effective at reducing RNT (worry and rumination), which are proven risk factors for multiple mental health problems. If the self-help app is effective at reducing worry and rumination, this will suggest that the intervention may be of value for long-term prevention and is worthy of further longer-term investigation.

Because this intervention is based on a smartphone app and is entirely self-help, without requiring support or therapist time, it has the ability to be scaled up to be widely available as a public health intervention. This work may thus contribute to large-scale effective preventions for young people, i.e., massive open online interventions (MOOI) [71], and has the potential to be hugely beneficial.

## Data Availability

Anonymised datasets arising from this trial will be made available after the primary outcomes are published to researchers and other groups via request to a data committee within the Consortium via the University of Exeter’s open access data system Open Research Exeter (ORE). The results will additionally be updated on ClinicalTrials.gov Identifier: NCT04950257. The ECoWeB consortium plans to communicate trial results through peer-reviewed open access publications and direct reports to TSC, sponsor, and participants.

## Abbreviations

ANCOVA: Analysis of Covariance
BIT: Behavioural Intervention Technology
CBT: Cognitive Behavioural Therapy
CPM: Component Proess Model of Emotion
CONSORT: Consolidated Standards of Reporting Trials
DMC: Data Monitoring Committee
EC: Emotional Competence
ECoWeB: Emotional Competence for Well-Being in Young Adults
FIML: full information maximum liklihood
GAD-7: Generalized Anxiety Disorder-7
ITT: intention-to-treat
MCID: minimum clinically important difference
MI: multiple imputations
MOOI: massive open online interventions
PHQ-9: Patient Health Questionnaire
PSWQ: Penn State Worry Questionnaire
RCT: randomised controlled trial
RNT: Repetetive Negative Thinking
RRS: Ruminative Response Scale
SD: Standard Deviation
TSC: Trial Steering Committee
UK: United Kingdom
WEMWBS: Warwick-Endinburgh Mental Well Being Scale

## Footnotes

1 Calculation based on the formula: n = f(α/2, β) × 2 × σ^2^ / (μ_1_ − μ_2_)^2^ where μ_1_ and μ_2_ are the mean outcome in the control and experimental group respectively, σ is the standard deviation, and f(α, β) = [Φ^− 1^(α) + Φ^− 1^(β)]^2^ Φ^− 1^ is the cumulative distribution function of a standardised normal deviate.
